# Two distinct mTORC2-dependent pathways converge on Rac1 to drive breast cancer metastasis

**DOI:** 10.1186/s13058-017-0868-8

**Published:** 2017-06-30

**Authors:** Meghan Morrison Joly, Michelle M. Williams, Donna J. Hicks, Bayley Jones, Violeta Sanchez, Christian D. Young, Dos D. Sarbassov, William J. Muller, Dana Brantley-Sieders, Rebecca S. Cook

**Affiliations:** 1 Department of Cancer Biology, Vanderbilt University School of Medicine, 2220 Pierce Avenue, Rm 749 Preston Research Building, Nashville, TN 37232 USA; 2 Department of Medicine, Vanderbilt University Medical Center, Nashville, TN 37232 USA; 3 Department of Molecular and Cellular Oncology, University of Texas MD Anderson Cancer Center, Houston, TX 77030 USA; 4 Department of Biochemistry, McGill University, Montreal, Quebec Canada

**Keywords:** mTOR, Rictor, Akt, Rac, Metastasis, Breast cancer, RhoGDI2, Protein kinase C, HER2, Mouse mammary tumor, Conditional knockout

## Abstract

**Background:**

The importance of the mTOR complex 2 (mTORC2) signaling complex in tumor progression is becoming increasingly recognized. *HER2-*amplified breast cancers use Rictor/mTORC2 signaling to drive tumor formation, tumor cell survival and resistance to human epidermal growth factor receptor 2 (HER2)-targeted therapy. Cell motility, a key step in the metastatic process, can be activated by mTORC2 in luminal and triple negative breast cancer cell lines, but its role in promoting metastases from *HER2-*amplified breast cancers is not yet clear.

**Methods:**

Because Rictor is an obligate cofactor of mTORC2, we genetically engineered Rictor ablation or overexpression in mouse and human *HER2*-amplified breast cancer models for modulation of mTORC2 activity. Signaling through mTORC2-dependent pathways was also manipulated using pharmacological inhibitors of mTOR, Akt, and Rac. Signaling was assessed by western analysis and biochemical pull-down assays specific for Rac-GTP and for active Rac guanine nucleotide exchange factors (GEFs). Metastases were assessed from spontaneous tumors and from intravenously delivered tumor cells. Motility and invasion of cells was assessed using Matrigel-coated transwell assays.

**Results:**

We found that Rictor ablation potently impaired, while Rictor overexpression increased, metastasis in spontaneous and intravenously seeded models of HER2-overexpressing breast cancers. Additionally, migration and invasion of *HER2*-amplified human breast cancer cells was diminished in the absence of Rictor, or upon pharmacological mTOR kinase inhibition. Active Rac1 was required for Rictor-dependent invasion and motility, which rescued invasion/motility in Rictor depleted cells. Rictor/mTORC2-dependent dampening of the endogenous Rac1 inhibitor RhoGDI2, a factor that correlated directly with increased overall survival in *HER2*-amplified breast cancer patients, promoted Rac1 activity and tumor cell invasion/migration. The mTORC2 substrate Akt did not affect RhoGDI2 dampening, but partially increased Rac1 activity through the Rac-GEF Tiam1, thus partially rescuing cell invasion/motility. The mTORC2 effector protein kinase C (PKC)α did rescue Rictor-mediated RhoGDI2 downregulation, partially rescuing Rac-guanosine triphosphate (GTP) and migration/motility.

**Conclusion:**

These findings suggest that mTORC2 uses two coordinated pathways to activate cell invasion/motility, both of which converge on Rac1. Akt signaling activates Rac1 through the Rac-GEF Tiam1, while PKC signaling dampens expression of the endogenous Rac1 inhibitor, RhoGDI2.

**Electronic supplementary material:**

The online version of this article (doi:10.1186/s13058-017-0868-8) contains supplementary material, which is available to authorized users.

## Background

Human epidermal growth factor receptor 2 (HER2) overexpression is a defining feature of approximately 25% of breast cancers and is associated with poor outcome [1]. While HER2-targeted therapies improve outcomes for patients with *HER2*-amplified breast cancers, resistance to HER2 inhibitors often occurs, underscoring the need for increased understanding of the signaling pathways required for HER2-mediated transformation and malignancy.

The intracellular mTOR kinase exists in two structurally and functionally distinct complexes, defined by the cofactors associated with mTOR, and by their relative sensitivity to rapamycin [2]. Specifically, Raptor is a required cofactor for rapamycin-sensitive mTORC complex 1 (mTORC1), which is activated downstream of phosphatidyl inositol-3 kinase (PI3K)/Akt and mediates cell growth, protein translation, and metabolism [3]. Rictor is a required cofactor for mTORC2, which controls cell survival, polarity, and cytoskeletal dynamics [4]. Control of cytoskeleton reorganization through regulation of the Rho family of GTPases was one of the first described functions of mTORC2 [5] and multiple studies indicate that mTORC2 regulates migration in various cell types including neutrophils, β-cells, endothelial cells, and primary mammary epithelial cells [6–9].

Given the potent role of motility in tumor progression, it is not surprising that mTORC2 has recently garnered interest for its potential role in cancer metastasis. Studies have shown that luminal breast cancer cells and basal-like breast cancer cells exploit Rictor-dependent mTOR signaling pathways to facilitate invasion and metastasis. For example, small interfering RNA (siRNA)-mediated Rictor knockdown has been shown to inhibit MCF7 (luminal) and MDA-MB-231 (basal-like) breast cancer cell migration [10, 11]. Rictor knockdown inhibits transforming growth factor beta (TGFβ)-mediated epithelial-to-mesenchymal transition (EMT) in basal-like breast cancer lines [12], and Prickle-dependent cell motility in the MDA-MB-231 model of breast cancer [13]. Additionally, mTOR-independent roles for Rictor in cancer cell migration have also been described. For example, in MDA-MB-231 or T47D (luminal) cells, Rictor was found to interact with protein kinase C (PKC)-ζ to control cancer cell metastasis [11]. However, the contribution of mTORC2 to migration of *HER2*-amplified breast cancer cells remains unclear. Notably, Rictor mRNA and protein levels are highest in *HER2*-amplified breast cancers, and correlate with decreased overall patient survival in clinical invasive breast cancer datasets [11, 14]. Rictor depletion from *HER2*-amplified human breast cancer cell lines or in genetically engineered transgenic mouse models of *HER2*-amplified breast cancer demonstrates the requirement for Rictor/mTORC2 signaling in tumor formation, tumor cell survival and resistance to HER2-targeted therapy [14]. Given the key role of Rictor in several stages of tumor progression, we were motivated to study the impact of Rictor/mTORC2 in migration, invasion and metastasis of HER2-driven breast cancers.

## Methods

### Mice

All animals were housed under pathogen-free conditions, and experiments were performed in accordance with AAALAC guidelines and with Vanderbilt University Institutional Animal Care and Use Committee approval. *Rictor* ^*FL/FL*^ mice [15] were kindly provided by Dr. Mark Magnuson (Vanderbilt University) and were inbred to FVB for >10 generations. *MMTV-NIC* mice (generated in FVB) have been previously described [16]. All analyses of *Rictor* ^*FL/FL*^ *X MMTV-NIC* mice were performed on age-matched siblings. For tail vein injections, 5 × 10^6^ SKBR3-scrambled short hairpin shRNA sequences (shScr), SKBR3-short hairpin RNA sequences against Rictor (shRictor), MDA-MB-361-shScr or MDA-MB-361-shRictor cells in 100 μL serum-free DMEM:F12 were delivered to isofluorane-anesthetized mice using a 27-g syringe needle. Freshly harvested *Rictor* ^*+/+*^ *NIC* and *Rictor* ^*FL/FL*^ *NIC* tumors were dissociated into single cell suspensions using C-tubes (Miltenyi) for mechanical dissociation, followed by digestion in collagenase A/hyaluronidase (Stem Cell Technologies) for 30 min., 0.05% trypsin-EDTA (Gibco) for 1 min., and DNase I (Stem Cell Technologies) for 10 min. Cells were passed through a 40-μm strainer, and 10^6^ cells were resuspended in 100 μl serum-free DMEM:F12 for delivery to isofluorane-anesthetized wildtype FVB female mice using a 27-g syringe needle.

### Histological analysis

Lungs were resected from mice and paraffin sections (5 μm) were stained with hematoxylin and eosin (Calbiochem). Immunohistochemical analysis (IHC) on paraffin-embedded sections was performed as described previously [17] using Rictor (Santa Cruz Biotechnologies) antibodies. Immunodetection was performed using the Vectastain kit (Vector Laboratories), according to the manufacturer’s directions.

### In situ Rac-guanosine triphosphate (GTP) assay

Formalin-fixed paraffin-embedded tumor sections were probed 1 h with glutathione-S transferase (GST)-PAK1 Binding Domain (PBD) (Millipore) diluted 1:50 in PBS. GST (lacking PBD) was used as a negative control. Samples were washed then probed with AF488-conjugated anti-GST (1:100), stained with 4',6-diamidino-2-phenylindole (DAPI), and mounted.

### Cell culture

BT474, MDA-MB-361, and SKBR3 cells were purchased in 2012 from American Type Culture Collection (ATCC) (cell identity verified by ATCC using genotyping with a Multiplex STR assay) and cultured at low passage in DMEM with 10% fetal calf serum. MCF10A and MCF10A-Rictor^ZFN^ cells (Sigma-Aldrich) were cultured in DMEM:F12 plus insulin (4 μg/mL), cholera toxin (1 μg/mL), epidermal growth factor (EGF) (100 ng/mL), hydrocortisone (2 μg/mL) and 5% horse serum and transduced with lentiviral HER2-internal ribosomal entry sequence (IRES)-RFP (GenTarget) and selected with 10 ug/mL blasticidin. MMTV-Neu tumor cells were a primary culture of mammary tumor cells derived from a virgin *MMTV-Neu* female mouse. Tumors were digested in 1X Collagenase A/Hyaluronidase solution (Stem Cell Technologies) for 30 min. at 37 °C, washed five times with serum-free medium, then plated on Matrigel-coated plates in serum-free DMEM:F12 plus insulin (4 μg/mL), EGF (10 ng/mL) and hydrocortisone (2 μg/mL). AZD5363, PP242, and lapatinib were purchased from SelleckChem. The in-solution Rac1 inhibitor was purchased from Calbiochem/Millipore. Adenoviral particles Ad.caRac1, Ad.PKCα, Ad. RFP, and Ad.Akt^DD^ were purchased from Vector Biolabs. *ARGHGDIB* siRNA’s were purchased from Sigma using the following siRNA IDs: SASI_Hs01_00125904 and SASI_Hs01_00125905.

### Generation of stable cell lines

Lentiviral shRNA-encoding pLKO plasmids harboring Rictor shRNA (#1853, referred to herein as shRictor.1, and #1854, referred to herein as shRictor.2); scrambled shRNA (#1864) and Raptor shRNA (#1857 referred to here as shRaptor.1 and #1858, shRaptor.2) were transfected into 293FT cells plus packaging vectors. Cultured medium containing viral load was used to infect. Cells were selected and maintained at low passage with puromycin (2 μg/mL). Mouse Rictor was subcloned from pCI-Avo3 (Jacinto et al. [5]) (Addgene #39210) using high-fidelity PCR and the following primers: forward 5′ CGC AAA TGG GCG GTA GGC TGT and reverse 5′GCT AGT TAT TGC TCA CGC C. Ends were blunted and then cloned into the blunted EcoRI site of pBABE-Puro. Retroviral particles were generated in 239 T cells transfected with pBABE vectors and pCL-Eco. Cultured medium containing viral load was filtered then added to MMTV-Neu cells. At 48 h after infection, MMTV-Neu cells were split 1:5 then selected and maintained with puromycin (2 μg/mL).

### Western blotting

Cells were homogenized in ice-cold lysis buffer (50 mM Tris pH 7.4, 100 mM NaF, 120 mM NaCl, 0.5% NP-40, 100 μM Na_3_VO_4_, 1X protease inhibitor cocktail (Roche)) and cleared by centrifugation (4 °C, 13,000 × *g*, 10 min.). Protein concentration was determined using bicinchoninic acid (BCA) assay (Pierce). Proteins separated by SDS-PAGE were transferred to nitrocellulose membranes. Membranes were blocked and probed with antibodies as described previously [18] using primary antibodies: α-actin (Sigma-Aldrich); Rictor (Santa Cruz); RhoGDI2 (Spring Bioscience); and the following from Cell Signaling Technologies (phospho-cocktail; AKT, P-Akt S473, P-Akt T308, S6, P-S6, and Raptor).

### Rac-GTP and Rac-guanine nucleotide exchange factor (Rac-GEF) pull down assays

GST-PBD beads (25 μg; Cytoskeleton #PAK02, resuspended to 1 μg/μL) were mixed with 750 μg cell lysate and rotated end-over-end for 1 h at 4 °C. Beads/lysate mixture was spun at 5000 rpm for 30 sec. Supernatant was removed, beads were washed in PBS, boiled in 13 μL denaturing sample buffer for 10 min., and supernatant was assessed by western analysis using primary antibody against Rac1 (BD Biosciences). The Active Rac-GEF Assay (Tiam1) kit (Cell Biolabs, Inc.) was used according to the manufacturer’s instructions. Briefly, cells at 70% confluence were harvested in the supplied 1X assay buffer, protein content assessed, and 750 μg protein lysate was incubated with 40 μL RacG15A-agarose bead slurry overnight at 4 °C. Beads were washed five times with assay buffer and then assessed by western analysis using the rabbit anti-Tiam1 antibody enclosed in the kit.

### Transwell migration/invasion assays

Breast cancer cells were serum-starved overnight, trypsinized, then 100,000 cells were seeded in the upper chamber of Matrigel-coated transwells in serum-free medium, with cells migrating towards the lower chamber in response to 10% serum or EGF (20 nM, R&D Systems). Cells on the lower side of the Matrigel-coated membrane were stained with 0.1% crystal violet after 24 h and enumerated as described previously [19].

### Statistics

Experimental groups were compared with a control group using Student’s unpaired, two-tailed *t* test. Multiple groups were compared across a single condition using one-way analysis of variance (ANOVA). Two-way ANOVA was used to compare the response of two agents combined to either of the single agents alone. *P* < 0.05 was used to define significant differences from the null hypothesis.

## Results

### Rictor loss decreases metastasis in a genetically engineered mouse model of HER2-driven breast cancer

**Fig. 1:**
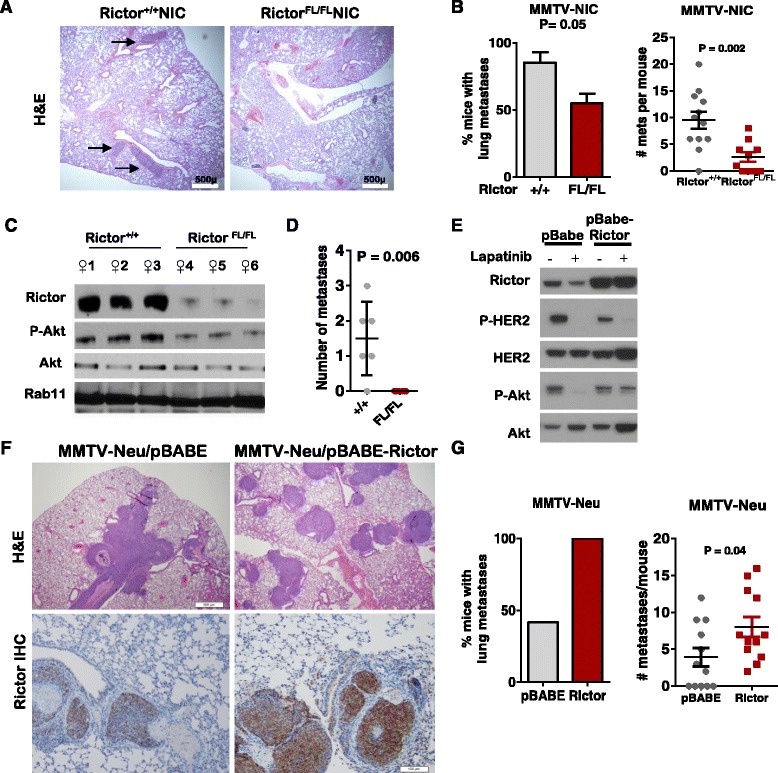
Rictor ablation decreases spontaneous and experimental metastasis in transgenic mammary tumors. **a** H&E staining of lungs from tumor-bearing *MMTV-NIC* mice. *Arrows* indicate metastases. Representative images are shown, original magnification, ×40. **b** *Left panel*: percentage of tumor-bearing mice harboring lung metastases. Non-tumor bearing mice were excluded from this analysis. *Error bars* represent the average of two experimental repeats. The first trial sample numbers were 7 *Rictor* ^*+/+*^ *NIC* and 5 *Rictor* ^*FL/FL*^ *NIC*. The second trial numbers were 5 *Rictor* ^*+/+*^ *NIC* and 4 *Rictor* ^*FL/FL*^ *NIC. Right panel*: number of lung metastases per tumor-bearing mouse was determined in H&E-stained sections through the lung. *Midlines* are the average number (± S.D.) of metastases/mouse. Each *data point* represents one tumor-bearing mouse; N = 12 *Rictor* ^*+/+*^ *NIC* and 9 *Rictor* ^*FL/FL*^ *NIC*. **c**-**d** Dissociated *Rictor* ^*+/+*^ *NIC* and *Rictor* ^*FL/FL*^ *NIC* tumors (N = 3) were partitioned for assessment by western analysis (**c**) or for venous delivery to wild-type (WT) FVB mice by (two recipient mice per donor). At 8 weeks, lungs were harvested from each recipient and assessed for metastatic lesions by whole-mount hematoxylin staining (**d**). *Midlines* are the average number (± S.D.) of metastases/mouse. Each *data point* represents one mouse. **e**-**g** *MMTV-Neu*-derived mouse mammary tumor cells retrovirally transduced with pBABE or pBABE-Rictor. **e** Cells were treated for 4 h with lapatinib, then assessed by western analysis using the antibodies indicated (*left*). **f** Venous delivery of cells to WT FVB recipient mice was followed 8 weeks later with examination of lungs from each recipient for metastatic lesions. Representative images are shown, original magnification × 40. *Upper panels*: histological sections. *Lower panels*: Rictor immunohistochemical analysis (*IHC*). **g** *Left panel*: percentage of mice harboring lung metastases, one trial, N = 12 per group. *Right panel*: number of lung metastases/mouse was determined as in **b**. *HER2* human epidermal growth factor receptor 2

Metastasis is a key behavior of tumor cells that contributes to breast cancer mortality. We used a transgenic mouse model of HER2-overexpressing breast cancer, *MMTV-NIC*, which enforces mammary-specific expression of oncogenic Neu, the rat HER2 homolog, from a bi-cistronic transgene harboring an internal ribosomal entry site (IRES) and a Cre recombinase expression cassette, resulting in formation of metastatic mammary tumors [20]. *MMTV-NIC* mice crossed with *Rictor* ^*FL/FL*^ mice resulted in elimination of Rictor specifically in Neu-expressing cells. Although Rictor loss decreases *MMTV-NIC* tumor formation [14], the impact of Rictor loss on *MMTV-NIC* tumor metastasis was not known. We assessed tumor-bearing *Rictor* ^*FL/FL*^ *NIC* mice at 28 days after initial tumor palpation (Fig. 1a), and found that only 55% (5/9) of tumor-bearing *Rictor* ^*FL/FL*^ *NIC* mice harbored lung metastases (Fig. 1b, left panel), as opposed to 86% of *Rictor* ^*+/+*^ *NIC* mice. Importantly, metastatic tumor burden per mouse was reduced in *Rictor* ^*FL/F*^ *NIC* mice versus controls (Fig. 1b, right panel). When the number of metastases per mouse was corrected for the volume of the tumor from which the metastatic lesions originated, the difference between *Rictor* ^*FL/FL*^ *NIC* and controls was diminished, although a trend towards reduced metastasis upon Rictor gene targeting was still observed (Additional file 1: Figure S1A). Low level expression of Rictor protein was detected in lung lesions from *Rictor* ^*FL/FL*^ *NIC* mice, albeit in only a small number of cells (Additional file 1: Figure S1B, arrows), suggesting that some metastatic tumor cells may have escaped Cre-mediated Rictor loss.

To confirm that Rictor loss decreases tumor metastasis, we dissociated three independently derived *Rictor* ^*FL/FL*^ *NIC* and *Rictor* ^*+/+*^ *NIC* primary tumors into single cell suspensions, then delivered equal numbers of cells to wild-type FVB mice via intravenous injection. Tumor cell suspensions were examined by western analysis to confirm reduction of Rictor protein levels in *Rictor* ^*FL/FL*^ *NIC* samples (Fig. 1c), although some Rictor expression remained, perhaps due to incomplete recombination at floxed *Rictor* alleles, or to an abundance of non-tumor cells in the tumor suspensions that would not be subjected to Cre-mediated recombination. Rictor loss diminished Akt phosphorylation at S473, the mTORC2 phosphorylation site, suggesting that mTORC2 activity was reduced upon Rictor loss in mammary tumors. At 8 weeks after delivery of tumor cells, mouse lungs were assessed for metastatic lesions. Lung metastases were identified in mice receiving *Rictor* ^*+/+*^ *NIC* tumor cells, while *Rictor* ^*FL/FL*^ *NIC* tumor cells failed to form metastatic lung lesions (Fig. 1d, N = 3 donor tumors per genotype times 2 recipients per donor tumor).

To determine the impact of increased Rictor expression on mammary tumor metastasis, we transduced primary mammary tumor cells derived from MMTV-Neu mice with pBABE retroviral particles encoding mouse Rictor, followed by puromycin selection. Western analysis confirmed Rictor overexpression in cells expressing pBABE-Rictor, but not in cells expressing empty pBABE (Fig. 1e). Rictor overexpression did not increase P-Akt levels under basal conditions, but prevented P-Akt inhibition following treatment of cells with lapatinib, an HER2 tyrosine kinase inhibitor. We found that intravenous delivery of MMTV-Neu/pBABE and MMTV-Neu/pBABE-Rictor cells generated lung metastases (Fig. 1f). Rictor overexpression in metastases derived from MMTV-Neu/pBABE-Rictor cells was confirmed by IHC. While MMTV-Neu/pBABE cells to wild-type FVB mice resulted in metastatic lung lesions in 41.7% of recipients, consistent with previous reports that MMTV-Neu mammary tumors metastasize to the lungs with a penetrance of approximately 30%, we found that 100% of MMTV-Neu/pBABE-Rictor recipients harbored lung metastases (Fig. 1g, left panel), and on average, harbored nearly twice as many metastatic lesion over recipients of MMTV-Neu/pBABE cells (Fig. 1g, right panel). These data suggest that Rictor promotes metastasis in transgenic mouse models of HER2-overexpressing breast cancer.

#### Metastasis of HER2-amplified breast cancer cells is reduced upon Rictor loss

**Fig. 2:**
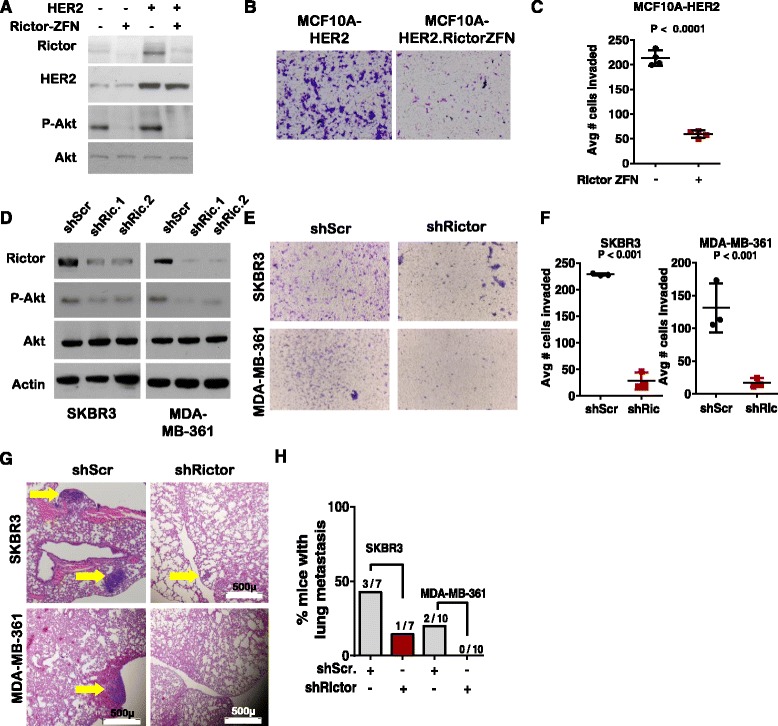
Rictor supports metastasis of human epidermal growth factor receptor 2 (*HER2*)-positive breast cancer cells. **a**-**c** MCF10A, MCF10A-HER2, MCF10A.Rictor^ZFN^ and MCF10A. Rictor^ZFN^-HER2 cells were assessed by western analysis **a** and for invasion through Matrigel-coated transwell filters (**b**, **c**). Cells were stained with crystal violet 24 h after plating in the upper well of transwell chambers, and imaged (representative images shown in (**b**). The numbers of cells on the bottom side of each transwell were counted in digital images by two independent investigators blinded to sample identity, and used to calculate the average number of cells invading to the lower side of each filter (**c**). Each *data point* represents the average value of three experimental replicates; *midline* represents the average of three biological repicates, *error bars* are the standard error. Student’s *t* test was used to assess significance. **d**-**f** SKBR3 and MDA-MB-361 cells stably expressing shRictor or shScr were assessed by western analysis (**d**) and for invasion through Matrigel-coated transwells (**e**, **f**), and quantitated as described in (**b**). **g**-**h** MDA-MB-361 and SKBR3 cells (5 × 10^6^) expressing scrambled short hairpin shRNA sequences (*shScr*) or short hairpin RNA sequences against Rictor (*ShRictor*) were delivered by tail vein injection to athymic Balb/C *nu/nu* female mice. Lungs harvested at 8 weeks post injection were assessed by histological analysis and imaged at × 40 (**g**); representative images shown, *arrows* indicate metastatic nodules). Mice were scored as positive or negative for metastatic nodules in the lung to determine the percentage of mice harboring metastatic nodules (**h**)

We next assessed genetic Rictor ablation in established models of human HER2-overexpressing breast cells. HER2 overexpression in parental MCF10A cells and genetically engineered Rictor-deficient MCF10A.Rictor^ZFN^ cells (generated using zinc finger nucleases (ZFNs) targeted to essential *RICTOR* gene sequences [9]) was used to generate MCF10A-HER2 and MCF10A.Rictor^ZFN^-HER2 cells. MCF10A.Rictor^ZFN^ and MCF10A.Rictor^ZFN^-HER2 cells lacked detectable Rictor expression and displayed impaired phosphorylation of Akt at S473, even in the context of HER2 overexpression, consistent with loss of mTORC2 activity (Fig. 2a). We measured cellular invasion through Matrigel-coated transwell filters using MCF10A-HER2 and MCF10A.Rictor^ZFN^-HER2 cells (Fig. 2b). Rictor loss reduced MCF10A-HER2 cell invasion by nearly 75% (Fig. 2c). These results were confirmed in Rictor-depleted human *HER2*-amplified breast cancer cell lines SKBR3 and MDA-MB-361 stably expressing Rictor shRNA sequences (shRictor). Western analysis confirmed Rictor knockdown by two independent sequences, which reduced Akt S473 phosphorylation (Fig. 2d). Invasion and migration of SKBR3 and MDA-MB-361 cells through Matrigel-coated transwell filters (Fig. 2e) was profoundly diminished in shRictor-expressing cells as compared to cells expressing control shRNA sequences (Fig. 2f).

The effect of Rictor knockdown in metastasis of *HER2*-amplified human breast cancer cells was examined by delivering 5 × 10^6^ SKBR3 or MDA-MD-361 cells expressing shScr and ShRictor to athymic Balb/C *(nu/nu*) mice via tail vein injection. Lungs harvested 8 weeks after tumor cell delivery were assessed in histological sections (Fig. 2g). These studies revealed lung metastases formed by shScr-expressing SKBR3 and MDA-MB-361 cells in 3/7 and 2/10 recipients, respectively (Fig. 2h), suggesting that these cell lines are not robust models of metastasis. Nonetheless, lung metastases were reduced upon Rictor knockdown, being detected in only one of the seven recipients of SKBR3-shRictor cells, and were not detected in any of the 10 recipients of MDA-MB-361-shRictor cells. These findings are consistent with the hypothesis that Rictor regulates metastasis in *HER2*+ breast cancer cells, and support previous studies demonstrating Rictor-dependent motility in other cancer cell lines, including estrogen receptor (ER)-α- breast cancer MCF7 cells and triple negative breast cancer (TNBC) MDA-MB-231 cells [10].

#### Rictor loss decreases Rac1-dependent cell migration and invasion in HER2-amplified breast cancer cells

**Fig. 3:**
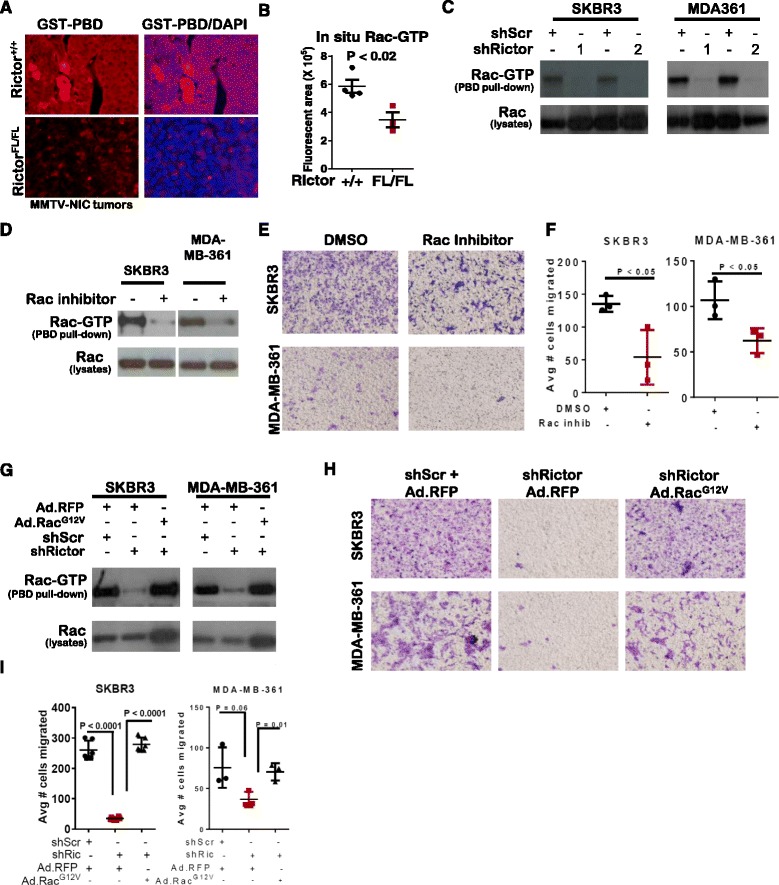
Rictor supports Rac-mediated migration and invasion of human epidermal growth factor receptor 2 (*HER2*)-positive breast cancer cells. **a**-**b** *In situ* detection of Rac-guanosine triphosphate (*GTP*) using indirect immunofluorescence via glutathione-S transferase-Pak binding domain (*GST-PBD*) in paraffin-embedded sections of *Rictor* ^*+/+*^ *NIC* and *Rictor* ^*FL/FL*^ *NIC* mammary tumors. GST-PBD binding is shown in *red*, while 4',6-diamidino-2-phenylindole (*DAPI*) counterstaining of nuclei is shown in *blue*. Representative images are shown (**a**), original magnification, ×200. Quantitation (**b**) shows the average fluorescent pixel area/group (*midlines*) ± S.D. and the average fluorescence in five random fields/sample (*points*); N = 3–4. **c** Whole cell lysates and PAK-PBD pull-downs from whole cell lysates were assessed by western analysis; N = 3 replicates. **d**-**f** Cells were treated with dimethyl sulfoxide (*DMSO*) or with Rac inhibitor (0.5 μM) and assessed by western analysis of whole cell lysates or PAK-PBD pulldowns (**d**) or assessed for migration through Matrigel-coated transwell filters 24 h after seeding, then stained with crystal violet for digital imaging (**e**); representative images shown) and quantitated (**f**). *Midlines* are the average number of cells on the lower side of the filter; *points* are average of three experimental replicates; experiments were repeated three times. **g**-**i** Cells expressing Ad.RFP or Ad.Rac^G12V^ were assessed by western analysis of whole cell lysates or PAK-PBD pulldowns (**g**), or for migration through Matrigel-coated transwell filters (**h**-**i**); N = 3. Quantitation is shown **i**. *Midlines* are the average number of migrating cells; *points* are replicates each assessed in triplicate

The small GTPase Rac1 potently activates cell motility in breast tumor cells, and is required in normal mammary epithelial cells downstream of mTORC2 [9]. Active, GTP-loaded Rac1 was measured using a PAK1 binding domain (PBD)-glutathione S transferase (GST) *in situ* binding assay [21] in *Rictor* ^*+/+*^ *NIC* and *Rictor* ^*FL/FL*^ *NIC* tumors, revealing abundant PBD-GST binding in *Rictor* ^*+/+*^ samples (Fig. 3a), but no reactivity of GST lacking the PBD in these same samples (Additional file 1: Figure S2A). Compared to what was seen in *Rictor* ^*+/+*^ *NIC* samples, PBD-GST binding was substantially reduced in *Rictor* ^*FL/FL*^ *NIC* tumors (Fig. 3b and Additional file 1: Figure S2B). PBD-GST pull-down assays similarly showed that Rac-GTP was decreased upon Rictor knockdown in SKBR3 and MDA-MB-361 cells (Fig. 3c), suggesting that Rictor/mTORC2 is required for Rac activation. Given the known role of Rac1 activity in cell migration of ER+ breast cancers and TNBCs, these results suggested that Rac1 may similarly be required for motility in *HER2*-amplified breast cancer cells. To test this, we used a pharmacological Rac inhibitor, which decreased PBD-GST pull-down of Rac1 in SKBR3 and MDA-MB-361 cells (Fig. 3d), and markedly reduced motility/invasion (Fig. 3e-f), suggesting that Rac activity is necessary in these cells for motility/invasion. To assess its role as an effector of Rictor/mTORC2, we restored Rac activity using expression of Rac(G12V), and active Rac1 mutant, which restored PBD-GST pull-down of Rac1 from shRictor cells (Fig. 3g) and fully rescued motility/invasion of shRictor-expressing cells (Fig. 3h-i, and Additional file 1: Figure S2C). These data suggest that Rac signaling downstream of Rictor/mTORC2 drives motility and invasion of *HER2*-amplified breast cancer cells.

#### mTORC2 signaling suppresses RhoGDI2 expression to enhance Rac1-mediated cell motility

**Fig. 4:**
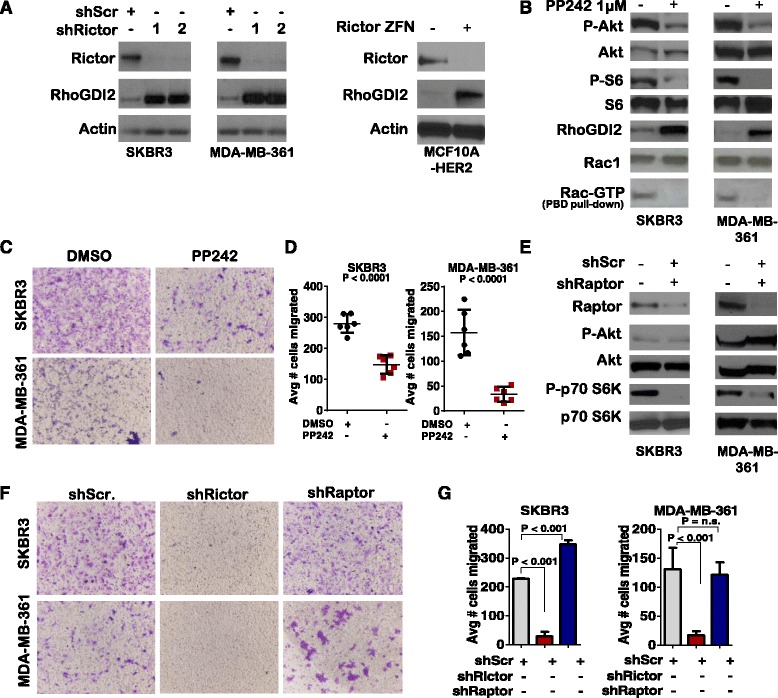
Rictor/mTORC2 activity downregulates Rho guanine nucleotide dissociation inhibitor (*RhoGDI2*) and stimulates cellular invasion. **a** Whole cell lysates were assessed by western analysis. **b**-**d** Cells were treated with dimethyl sulfoxide (*DMSO*) or with PP242 (1 μM) for 4 h. Whole cell lysates or Pak-Pak binding domain pulldowns from whole cell lysates were assessed by western analysis (**b**) or assessed for migration through Matrigel-coated transwell filters 24 h after seeding, then stained with crystal violet for digital imaging (**c**); representative images shown) and quantitated (**d**). *Midlines* are the average number of cells on the lower side of the filter; *points* are average of three experimental replicates, experiments were repeated three times. **e** Cells stably expressing scrambled short hairpin shRNA sequences (*shScr*) or short hairpin RNA sequences against Raptor (*shRaptor*) were assessed by western analysis. **f**-**g** Cells seeded in the upper chambers of Matrigel-coated transwell filters were assessed for invasion and migration at 24 h after seeding using crystal-violet-staining *and enumeration* (**g**) * as described in* (***d***)*; N = 4. HER2* human epidermal growth factor receptor 2, *GTP* guanine triphosphate, *shRictor* short hairpin RNA sequences against Rictor

To identify Rictor-dependent signaling pathways that contribute to Rac activation in *HER2*-amplified breast cancers, we examined the impact of Rictor knockdown on the expression of an endogenous inhibitor of Rac activity, Rho guanine nucleotide dissociation inhibitor 2 (RhoGDI2), based on previous observations that Rictor causes RhoGDI2 downregulation in mouse embryonic fibroblasts (MEFs) [22]. While RhoGDI2 expression was detected only at low levels in SKBR3 and MDA-MB-361 cells expressing shScr, we observed RhoGDI2 accumulation in cells expressing shRictor (Fig. 4a). These results were confirmed in Rictor-deficient MCF10A.Rictor^ZFN^-HER2 cells. While these findings are consistent with previous observations made in MEFs, the previous study demonstrated clearly and thoroughly that in MEFs, kinase activity of mTORC2 was not required for RhoGDI2 downregulation. However, we found that mTORC2 kinase inhibition using the mTORC1/mTORC2 dual kinase inhibitor PP242 permitted RhoGDI2 accumulation in SKBR3 and MDA-MB-361 cells, and resulted in decreased Rac-GTP (Fig. 4b). Importantly, PP242 decreased migration of SKBR3 and MDA-MB-361 cells though transwell filters (Fig. 4c-d). Thus, our findings suggest that in *HER2*-amplified breast cancer cells, blockade of mTOR kinase activity stabilizes RhoGDI2.

Because PP242, like other mTOR kinase inhibitors, inhibits both mTORC1 and mTORC2, it is possible that blockade of mTORC1 accounts partially or in full for impaired dampening of RhoGDI2 expression and decreased Rac1 activation. Therefore, we assessed the impact of selective mTORC1 inhibition on RhoGDI2 expression and Rac1 activity. Although the mTOR inhibitor rapamycin and the related rapalogues exhibit greater selectivity for mTORC1 over mTORC2, accumulating evidence suggests that rapalogues indirectly block mTORC2 activity in many cell types, including mammary epithelial cells. To ensure selective mTORC1 inhibition, we used shRNA sequences directed against Raptor, the mTORC1 obligate cofactor. Previous studies demonstrate that Raptor knockdown blocks mTORC1 signaling. Consistent with this, Raptor knockdown in SKBR3 and MDA-MB-361 cells, which was confirmed by western analysis, resulted in decreased phosphorylation of the mTORC1 substrate p70 S6 Kinase (p70^S6K^) (Fig. 4e). Phosphorylation of Akt at Ser473, the mTORC2 phosphorylation site, was unaffected by Raptor knockdown, confirming selective inhibition of mTORC1 signaling by Raptor knockdown. Raptor knockdown did not decrease invasion/migration of cells through Matrigel-coated transwell filters, as opposed to what was seen with Rictor knockdown (Fig. 4f-g). Further, RhoGDI2 expression was unaffected by Raptor knockdown in SKBR3 cells (Additional file 1: Figure S3). These results suggest that mTORC2 signaling is distinctly important for supporting migration in *HER2*-amplified breast cancer cells, due at least in part to regulation of RhoGDI2 levels.

#### RhoGDI2 suppression increases tumor cell motility and decreases metastasis-free survival

**Fig. 5:**
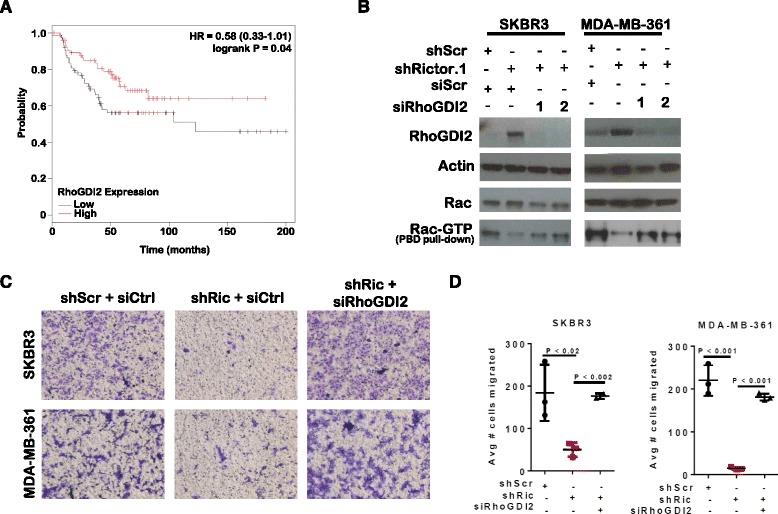
Decreased Rho guanine nucleotide dissociation inhibitor (*RhoGDI2*) levels correlate with increased cell motility and poor outcome in human epidermal growth factor receptor 2 (HER2) + breast cancers. **a** RhoGDI2/*ARHGDIB* mRNA expression levels were assessed in HER2+ breast tumors curated in publicly available breast cancer expression array datasets, using Km Plotter software (kmplot.org) to identify Affymetrix probe 1555812_a_at (*ARHGDIB*/RhoGDI2; N = 150). Samples were separated at the median level RhoGDI2/*ARHGDIB* expression, and plotted against metastasis-free survival for each sample in the Kaplan-Meier curve shown. **b**-**d** Cells were transiently transfected with siRNA sequences against RhoGDI2 or non-specific control siRNA. Whole cell lysates or Pak-Pak binding domain pull-downs from whole cell lysates were assessed by western analysis (**b**). Cells were assessed for invasion and migration through Matrigel-coated transwells 24 h after seeding by crystal violet staining. Representative images are shown (**c**). Quantitation of cells on the lower side of the filter is shown (**d**). *Midlines* are the average of three repeats and *points* are replicates, with each assessed in duplicate. Student’s *t* test was used to assess significance. *HR* hazard ratio, *shScr* scrambled short hairpin shRNA sequences, *GTP* guanosine triphosphate, *shRictor* short hairpin RNA sequences against Rictor, *siCtrl* small interfering control

To determine the potential clinical impact of RhoGDI2 on metastasization of *HER2*-amplified breast tumors, we examined mRNA expression of the gene encoding RhoGDI2, *ARHGDIB*, in publicly available HER2+ breast tumor datasets (N = 150, Kmplot.com). Upon bifurcating the sample population at the median into tumors with high versus low *ARHGDIB* expression, we found that patients whose tumors expressed higher *ARHGDIB* experienced greater metastasis-free survival as compared to patients with lower *ARHGDIB* (Fig. 5a). Interestingly, similar correlation was seen between high *ARHGDIB* expression and lengthened survival in a larger breast cancer dataset (N = 1660) that included all breast cancer molecular subtypes (Additional file 1: Figure S4), suggesting that RhoGDI2/*ARHGDIB* may be a key factor in breast tumor cell metastasis beyond the scope of *HER2*-amplified breast cancers. Depletion of RhoGDI2 in shRictor-expressing cells using RhoGDI2 siRNA (Fig. 5b) restored Rac1-GTP levels to what was seen in cells expressing shScr, and fully rescued cell migration of Rictor-depleted SKBR3 and MDA-MB-361 cells (Fig. 5c-d). These data suggest that Rictor negatively regulates RhoGDI2, permitting Rac1 activation and enhancing migration of *HER2*-amplified breast cancer cells.

#### Akt partially rescues migration and invasion of HER2-amplified breast cancer cells lacking Rictor

**Fig. 6:**
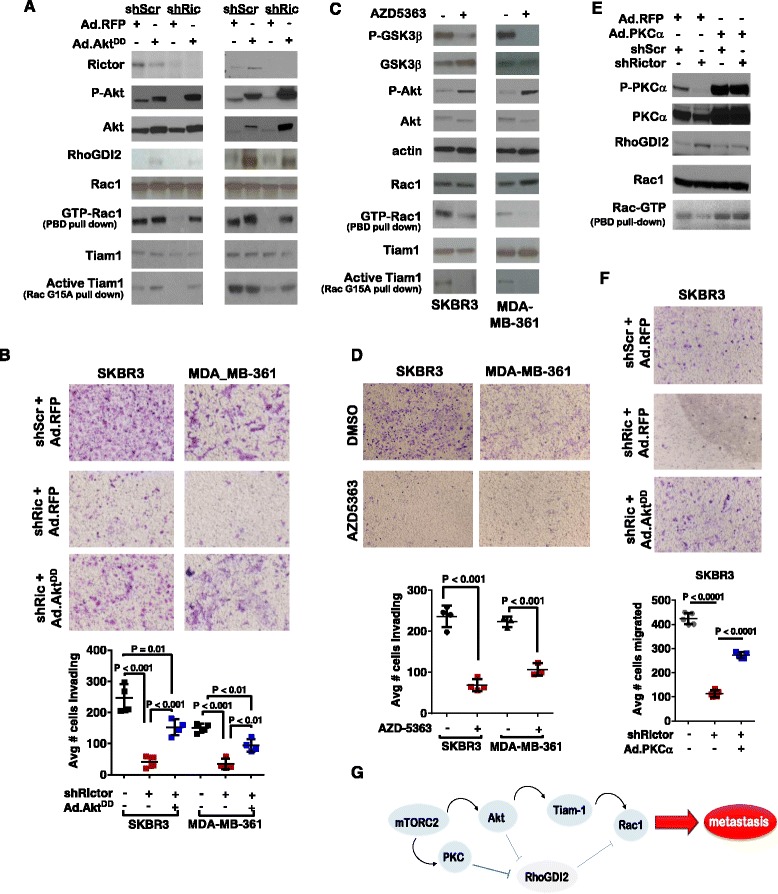
Akt restoration partially rescues Rictor-mediated defects in cell migration and invasion. **a**-**b** Cells expressing scrambled short hairpin shRNA sequences (*shScr*) or short hairpin RNA sequences against Rictor (*shRic*) were infected with Ad.RFP or Ad.Akt^DD^. Whole cell lysates, Pak binding domain (PBD) pulldowns from whole cell lysates, or Rac^G15A^ pulldowns from whole cell lysates were assessed by western analysis (**a**). Cells were assessed for their ability to invade through Matrigel-coated transwell filters at 24 hours after seeding (**b**). Cells on the lower side of the transwell filter were stained with crystal violet (representative images shown (**b** (*upper panel*)) and counted in digital images (**b** (*lower panel*)). Each *data point* represents the average value of three experimental replicates; *midline* represents the average of N = 4 repeats per condition, *error bars* are the standard error. Student’s *t* test used to assess significance. **c**-**d** Cells were treated with the Akt inhibitor AZD5365 (1 μM). Whole cell lysates, PBD pulldowns from whole cell lysates, or Rac^G15A^ pulldowns from whole cell lysates were assessed by western analysis (**c**). Cells were assessed for their ability to invade through Matrigel-coated transwell filters at 24 h after seeding (**d**). Cells on the lower side of the transwell filter were stained with crystal violet (representative images shown in **d** (*upper panel*) and counted in digital images (**d** (*lower panel*)) as described in (**b**). **e**-**f** Cells expressing shScr or shRictor were infected with Ad.RFP or Ad.PKCα. Whole cell lysates or PBD pulldowns from whole cell lysates were assessed by western analysis (**e**). Cells were assessed for their ability to invade through Matrigel-coated transwell filters at 24 h after seeding (**f**). Cells on the lower side of the transwell filter were stained with crystal violet (representative images shown in **f** (*upper panel*) and counted in digital images (**f** (*lower pane*l)) as described in (**b**). (**g**). Proposed model of how mTORC2 controls cell motility and metastasis of HER2-overexpressing breast cancers. *RhoGDI2* Rho guanine nucleotide dissociation inhibitor, *PKC* protein kinase C

Akt is a direct substrate of mTORC2 and increases motility in many cell types [23], supporting the hypothesis that mTORC2-mediated Akt activity may be required for RhoGDI2 dampening and subsequent Rac1 activation. We rescued Akt signaling in shRictor-expressing cells using expression of an Akt^T308D/S473D^ mutant (Ad.Akt^DD^) [24], (Fig. 6a). Interestingly, Akt^DD^ partially rescued Rac-GTP levels in Rictor-depleted cells, but had no impact on expression levels of RhoGDI2. Despite having no impact on RhoGDI2 expression, Akt^DD^ partially rescued invasion and motility of Rictor-depleted cells (Fig. 6b and Additional file 1: Figure S6). Therefore, we investigated activators of Rac1, a family of factors called guanine nucleotide exchange factors (GEFs), that might be activated downstream of Akt. Previous expression profiling studies revealed that Tiam1 was among the most highly expressed Rac-GEFs in *HER2*-induced mouse mammary tumors [25]. To determine if Tiam1 activity was affected by Rictor knockdown, we used nucleotide-free RacG15A beads to pull down active Rac-GEFs from SKBR3 and MDA-MB-361 cells expressing shRictor. We found active Tiam1 was pulled down from cells expressing shScr, but not from cells expressing shRictor. Further, expression of Akt^DD^ increased Tiam1 pulled down from Rictor-depleted cells. In contrast to the gene encoding the Rac inhibitor RhoGDI2, which correlated with a favorable outcome in breast cancer patients, increased expression of *TIAM1* mRNA in the METABRIC breast cancer microarray dataset that correlated with worse outcome for patients with breast cancer (Additional file 1: Figure S5), consistent with its potential role in regulating tumor metastasis.

These data suggest that Rictor/mTORC2 engages Akt signaling to activate Rac1 in SKBR3 and MDA-MB-361 cells, but not through downregulation of RhoGDI2, but rather through activation of Tiam-1. This hypothesis was confirmed using the ATP-competing Akt inhibitor AZD5363, which increases Akt phosphorylation while blocking Akt enzymatic activity. AZD5363 decreased phosphorylation of the Akt substrate glucose synthase kinase (GSK)-3β, decreased active Rac, and decreased active Tiam1 in SKBR3 and MDA-MB-361 cells (Fig. 6c), and substantially reduced cell motility/invasion (Fig. 6d). These results suggest that Rictor-mediated Akt signaling increases Tiam1 activation to support Rac1 activity.

To determine if another mTORC2 effector might be responsible for RhoGDI2 downregulation, we investigated protein kinase C (PKC)α, a direct substrate of mTORC2. Previous studies have shown that PKCα interacts directly with RhoGDI2, causing RhoGDI2 phosphorylation and downregulation. We found that phosphorylation of PKCα was abolished in Rictor-depleted cells (Fig. 6e). We used PKCα overexpression to increase PKCα activity and phosphorylation, resulting in RhoGDI2 downregulation in Rictor-depleted cells, and at the same time, increased Rac-GTP. Further, rescue of PKCα activity increased invasion and motility of Rictor-depleted cells through Matrigel-coated transwell filters (Fig. 6f). These studies suggest that mTORC2 coordinates two signaling pathways, Akt and PKCα, which destabilize the Rac inhibitor RhoGDI2, while using Akt to activate the Rac-GEF Tiam-1, to achieve maximal Rac activation and cell motility, supporting metastasis in *HER2*-amplified breast cancers (Fig. 6g).

## Discussion

In our previous studies, restoration of Akt signaling was sufficient to fully restore cell survival downstream of Rictor [14]. However, we show here that constitutive Akt signaling only partially rescued the invasion defects caused by Rictor loss. Several lines of evidence suggest that cancer cells exploit Rictor-dependent signaling pathways to facilitate invasion and metastasis. For example, siRNA-mediated Rictor depletion decreases migration of MCF7 and MDA-MB-231 cells [10, 11]. In gliomas, Rictor overexpression promotes mTORC2 activity and tumor cell growth and motility [26, 27]. Herein, we report that *HER2*-amplified breast cancer cell lines and tumors significantly decrease Rac1 activity upon Rictor knockdown. Recent studies in MEFs showed that Rictor suppresses expression of RhoGDI2, an endogenous Rac1 inhibitor and suppressor of metastasis, thus facilitating Rac1-mediated cell motility [22]. Results shown herein are in agreement with this observation, providing the first validation of this mechanism (Rictor/mTORC2 suppression of RhoGDI2 to activate Rac) in *HER2*-amplified breast cancer cell migration/metastasis.

Although these data are the first (to our knowledge) linking mTORC2/Rictor to Rac1-mediated invasion and metastasis in spontaneous breast cancer models, Rictor-to-Rac1 signaling has been previously identified in cancers originating from other tissues [28–30], and previous studies have shown that *HER2*-amplified breast cancer cells use p120 Catenin-to-Rac1 signaling to promote breast cancer metastasis [31]. These previous reports, taken together with data shown here, highlight the numerous signaling pathways that can converge on Rac1 to promote dissemination of cancer cells [9]. We show evidence that the endogenous Rac inhibitor RhoGDI2 is an important molecular brake for restraining Rac activity. Since RhoGDI2 expression levels correlated significantly with outcome in breast cancer datasets, it is possible that RhoGDI2 downregulation could be used as a predictor of aggressive versus benign disease, although this is a hypothesis that has not yet been tested.

Although Rictor-mediated RhoGDI2 downregulation has been previously reported in MEFs [22], those studies showed that this is an mTORC2-independent role for Rictor. However, we have shown here that inhibition of mTOR kinase activity using the small molecular weight compound PP242 caused a similar accumulation of RhoGDI2, diminution of Rac-GTP, and blockade of tumor cell motility as was seen upon Rictor knockdown. This suggests that, unlike what occurs in MEFs, *HER2*-amplified breast cancer cells use Rictor within the context of catalytically active mTORC2 to downregulate RhoGDI2, allowing Rac1 activation and cell motility.

Efficient metastatic progression relies on a coordinated series of steps involving cell motility and invasion, in addition to cell survival and proliferation [32]. While we show that Rictor loss decreases metastatic ability of HER2-driven breast tumors and that Rictor/mTORC2 directly controls cell migration, we have not ruled out the potential impact Rictor loss may have on survival of disseminated tumor cells and how this may affect seeding/establishment of overt metastases in our mouse models of metastasis used for this study. Since previous studies indicate that mTORC2 is required for survival of *HER2*-amplified breast cancer cells [10, 14], it is likely that the reduced metastases seen upon knockdown of Rictor are due to both a reduction in tumor cell survival and a reduction in tumor cell motility. However, tumor cell survival in Rictor-deficient *HER2*-amplified breast cancer cells was fully rescued by re-activation of Akt signaling. In contrast, we show herein that Akt re-activation only partially rescued invasion and migration of *HER2*-amplified breast cancer cells lacking Rictor. Thus, Akt signaling is necessary and sufficient for mTORC2-mediated tumor cell survival, but not for mTORC2-mediated cell motility.

## Conclusion

These studies demonstrate the requirement for mTORC2 signaling in *HER2*-amplified breast cancer metastasis through Rac1. Importantly, these studies demonstrate that Rictor/mTORC2 suppresses the Rac inhibitor RhoGDI2, while activating the Rac-GEF Tiam-1, through two parallel pathways that cooperate to potently activate Rac-dependent cell migration and invasion. These findings support growing literature describing the many pathways that activate mTORC2 and those that are activated by mTORC2. Importantly, evidence of the key roles played by mTORC2 in cancer formation, progression, and metastasis is expanding rapidly.

## Additional file

Additional file 1: Figure S1.
** a** The average metastatic potential index (numbers of metastases/tumor volume x 1000) is shown for individual mouse (by each bar) and for each group (below each genotype). *P* = not significant, Student’s *t* test. **b** Rictor IHC on lungs from tumor-bearing *MMTV-NIC* mice. *Black arrows* indicate Rictor + tumor cells. Representative images are shown, original magnification, ×400. **Figure S2. a** GST lacking the PBD domain does not produce staining. **b** Low-power image showing PBD binding to Rictor^+/+^NIC tumors, but not Rictor^FL/FL^NIC tumors. **c** SKBR3-shScr and SKBR3-shRictor were transduced with Ad.Rac^G12V^ or with Ad.RFP and assessed for invasion towards EGF at 24 h after seeding. Shown is the average of N = 5 experiments. *Bars* average ± S.D. Student’s *t* test **Figure S3.** SKBR3-shScr, SKBR3-shRictor, and SKBR3-shRaptor cells were assessed by western analysis **Figure S4.** RhoGDI2 mRNA expression levels were assessed in breast tumors (all subtypes) using publicly available breast cancer expression array datasets and Km Plotter software (kmplot.org). Affymetrix probe 1555812_a_at (*ARHGDIB*/RhoGDI2) in breast cancer samples (N = 1660) was used. Samples were separated at the median level RhoGDI2/*ARHGDIB* expression, and plotted against metastasis-free survival for each sample in the Kaplan-Meier curve shown. **Figure S5.** *TIAM1* mRNA expression levels were assessed in breast tumors (all subtypes) using publicly available breast cancer expression array datasets and cBio software (cbio.org). The METABRIC data set (N = 2509) was used. Samples with *TIAM1* expression >1.5 S.D. from the mean for all tumors in the group, combined with tumors gene amplified for *TIAM1* (for a total of 6% of the 2509 tumors) were plotted against overall survival for each sample. Log-rank test was calculated by cBio software. **Figure S6.** SKBR3-shScr and SKBR3-shRictor were transduced with Ad.Akt^DD^ or with Ad. RFP. and assessed for invasion towards EGF at 24 h after seeding. Shown is the average of N = 5 experiments. *Bars* average ± S.D. Student’s *t* test. (PPTX 2532 kb)

## Abbreviations

ANOVA: Analysis of variance
ATCC: American Type Culture Collection
BCA: Bicinchoninic acid
DAPI: 4',6-Diamidino-2-phenylindole
DMEM: Dulbecco’s modified Eagle’s medium
DMSO: Dimethyl sulfoxide
EGF: Epidermal growth factor
EMT: Epithelial mesenchymal transition
ER: Estrogen receptor
GEFs: Guanine nucleotide exchange factors
GST-PBD: Glutathione-S transferase-Pak binding domain
GTP: Guanosine triphosphate
H&E: Hematoxylin and eosin
HER2: Human epidermal growth factor receptor 2
IHC: Immunohistochemical analysis
IRES: Internal ribosomal entry site
MEF: Mouse embryonic fibroblast
mTORC: mTOR complex
NIC: Neu-internal ribosomal entry site-Cre
PI3K: Phosphatidyl inositol-3 kinase
PBS: Phosphate-buffered saline
PKC: Protein kinase C
RhoGDI2: Rho guanine nucleotide dissociation inhibitor
shRaptor: Short hairpin RNA sequences against Raptor
shRictor: Short hairpin RNA sequences against Rictor
shScr: Scrambled short hairpin shRNA sequences
siRNA: Small interfering RNA
TGF-β: Transforming growth factor beta
TNBC: Triple negative breast cancer
