# Supplementary material for: Two distinct mTORC2-dependent pathways converge on Rac1 to drive breast cancer metastasis
Source: Breast Cancer Res. 2017 Jun 30;19:74. doi: 10.1186/s13058-017-0868-8 (PMC5493112; doi:10.1186/s13058-017-0868-8)
Supplement: Additional file 1: Figure S1. — a The average metastatic potential index (numbers of metastases/tumor volume x 1000) is shown for individual mouse (by each bar) and for each group (below each genotype). P = not significant, Student’s t test. b Rictor IHC on lungs from tumor-bearing MMTV-NIC mice. Black arrows indicate Rictor + tumor cells. Representative images are shown, original magnification, ×400. Figure S2. a GST lacking the PBD domain does not produce staining. b Low-power image showing PBD binding to Rictor+/+NIC tumors, but not RictorFL/FLNIC tumors. c SKBR3-shScr and SKBR3-shRictor were transduced with Ad.RacG12V or with Ad.RFP and assessed for invasion towards EGF at 24 h after seeding. Shown is the average of N = 5 experiments. Bars average ± S.D. Student’s t test Figure S3. SKBR3-shScr, SKBR3-shRictor, and SKBR3-shRaptor cells were assessed by western analysis Figure S4. RhoGDI2 mRNA expression levels were assessed in breast tumors (all subtypes) using publicly available breast cancer expression array datasets and Km Plotter software (kmplot.org). Affymetrix probe 1555812_a_at (ARHGDIB/RhoGDI2) in breast cancer samples (N = 1660) was used. Samples were separated at the median level RhoGDI2/ARHGDIB expression, and plotted against metastasis-free survival for each sample in the Kaplan-Meier curve shown. Figure S5. TIAM1 mRNA expression levels were assessed in breast tumors (all subtypes) using publicly available breast cancer expression array datasets and cBio software (cbio.org). The METABRIC data set (N = 2509) was used. Samples with TIAM1 expression >1.5 S.D. from the mean for all tumors in the group, combined with tumors gene amplified for TIAM1 (for a total of 6% of the 2509 tumors) were plotted against overall survival for each sample. Log-rank test was calculated by cBio software. Figure S6. SKBR3-shScr and SKBR3-shRictor were transduced with Ad.AktDD or with Ad. RFP. and assessed for invasion towards EGF at 24 h after seeding. Shown is the average of N = 5 experiments. Bars avera [file 13058_2017_868_MOESM1_ESM.pptx]

## Slide 1
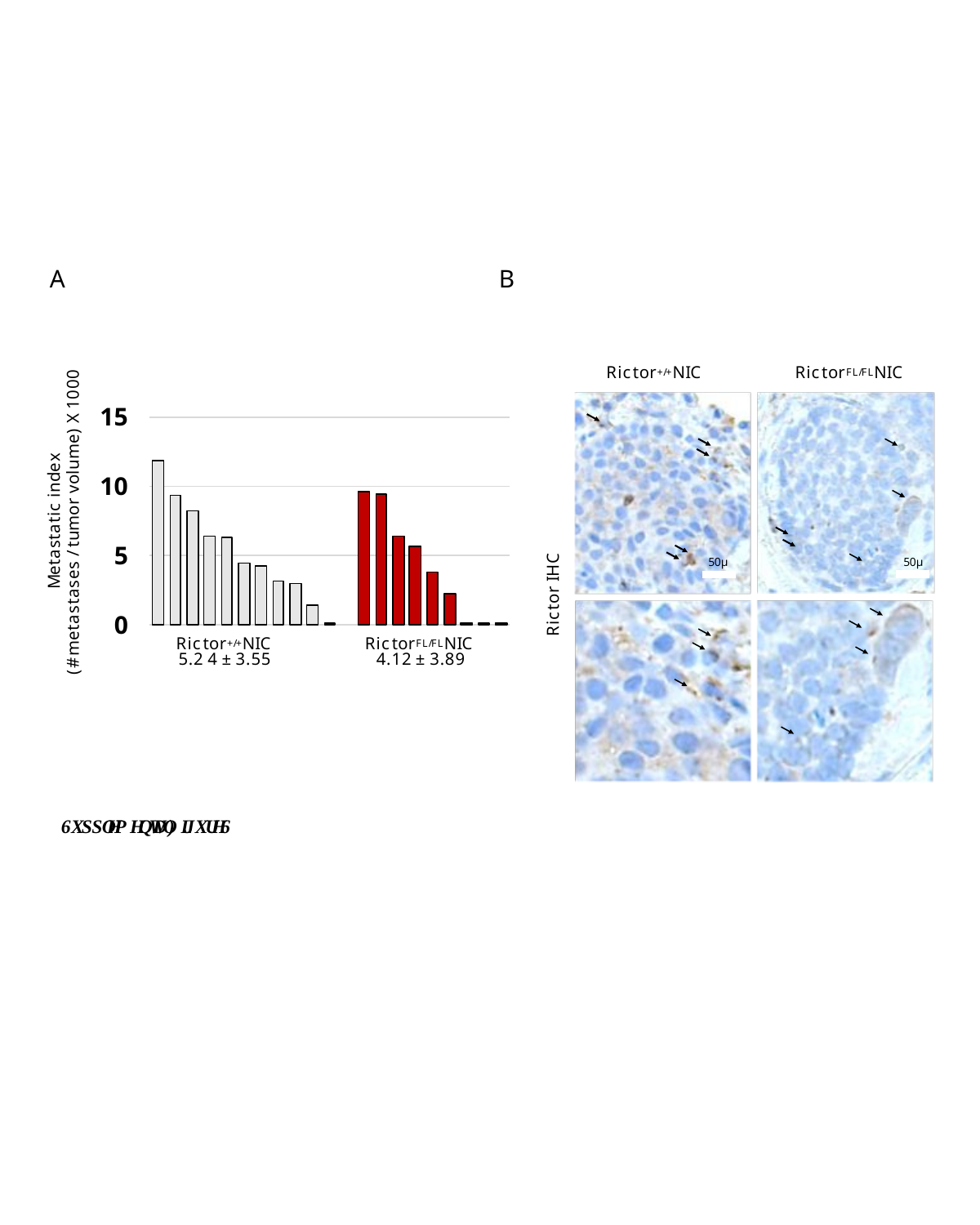

## Slide 2
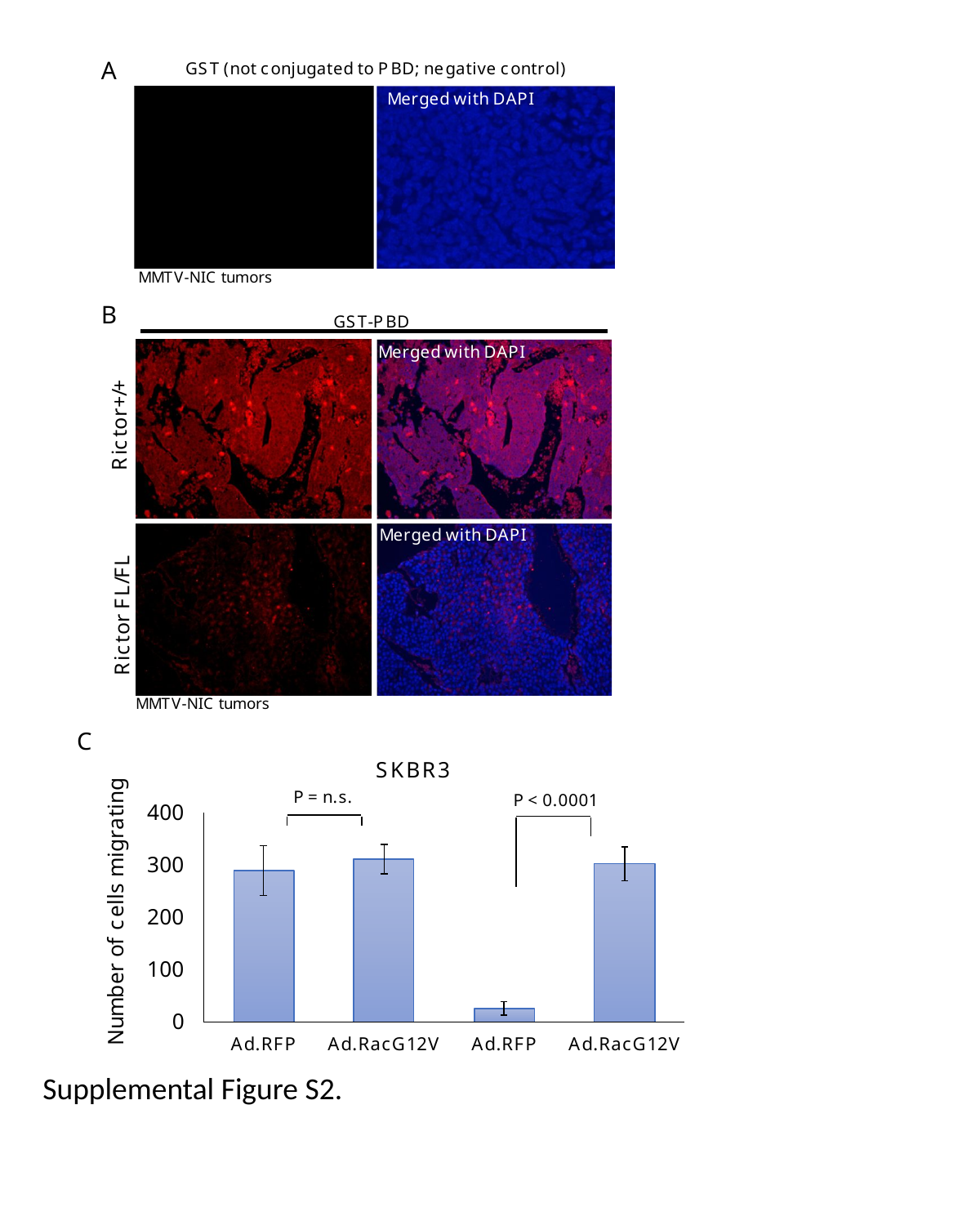

C
Supplemental Figure S2.

## Slide 3
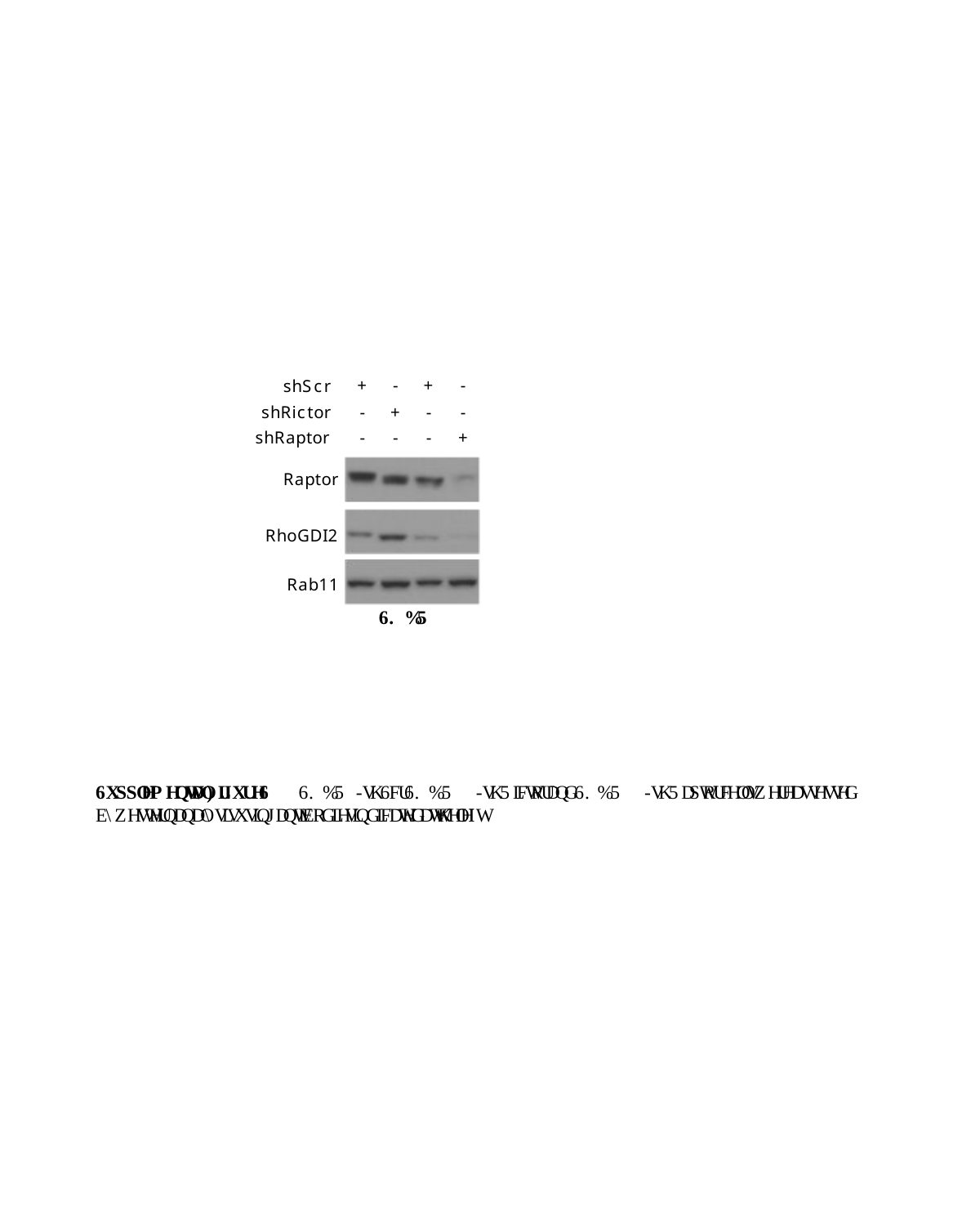

## Slide 4
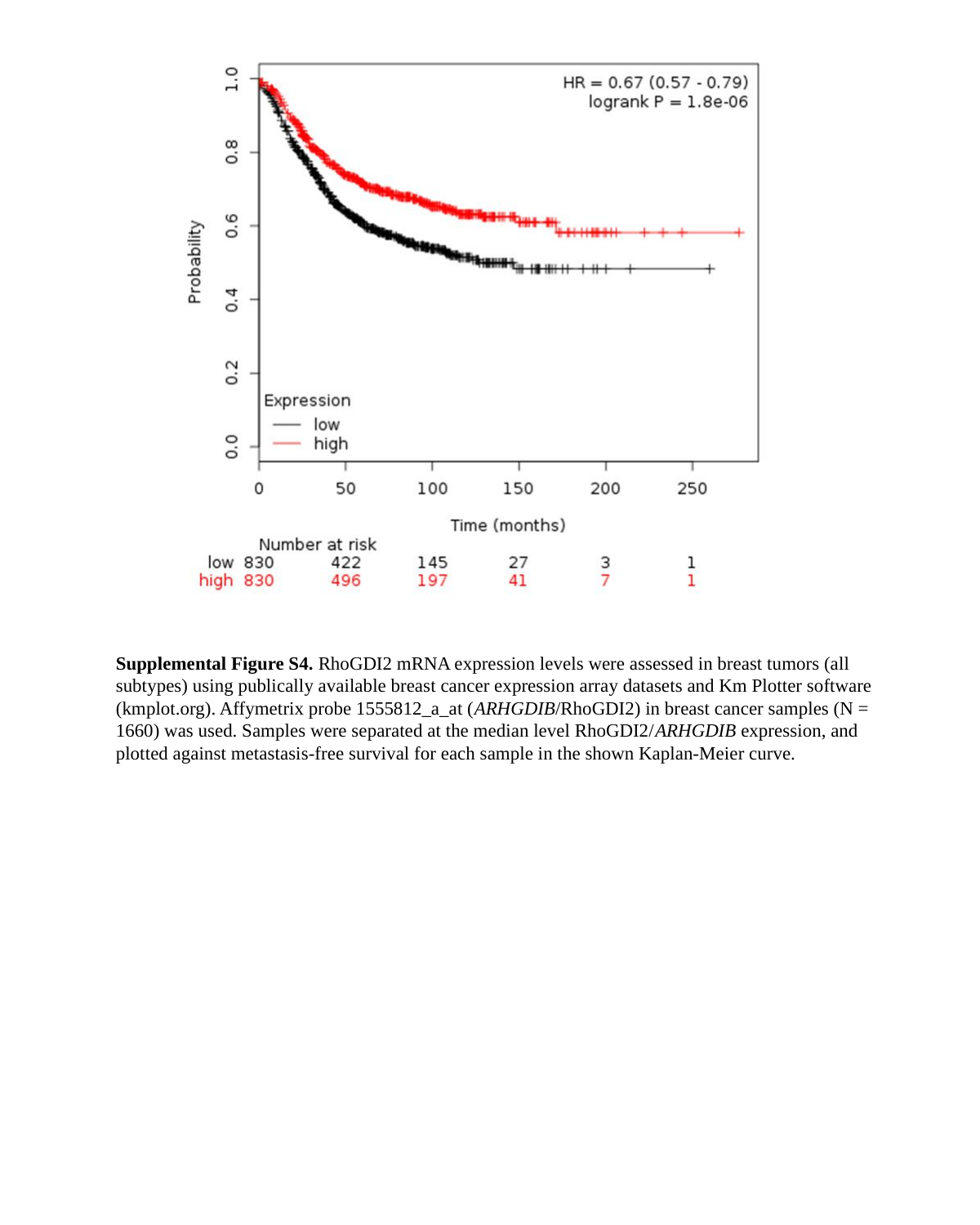

Supplemental Figure S4. RhoGDI2 mRNA expression levels were assessed in breast tumors (all subtypes) using publically available breast cancer expression array datasets and Km Plotter software (kmplot.org). Affymetrix probe 1555812_a_at (ARHGDIB/RhoGDI2) in breast cancer samples (N = 1660) was used. Samples were separated at the median level RhoGDI2/ARHGDIB expression, and plotted against metastasis-free survival for each sample in the shown Kaplan-Meier curve.

## Slide 5
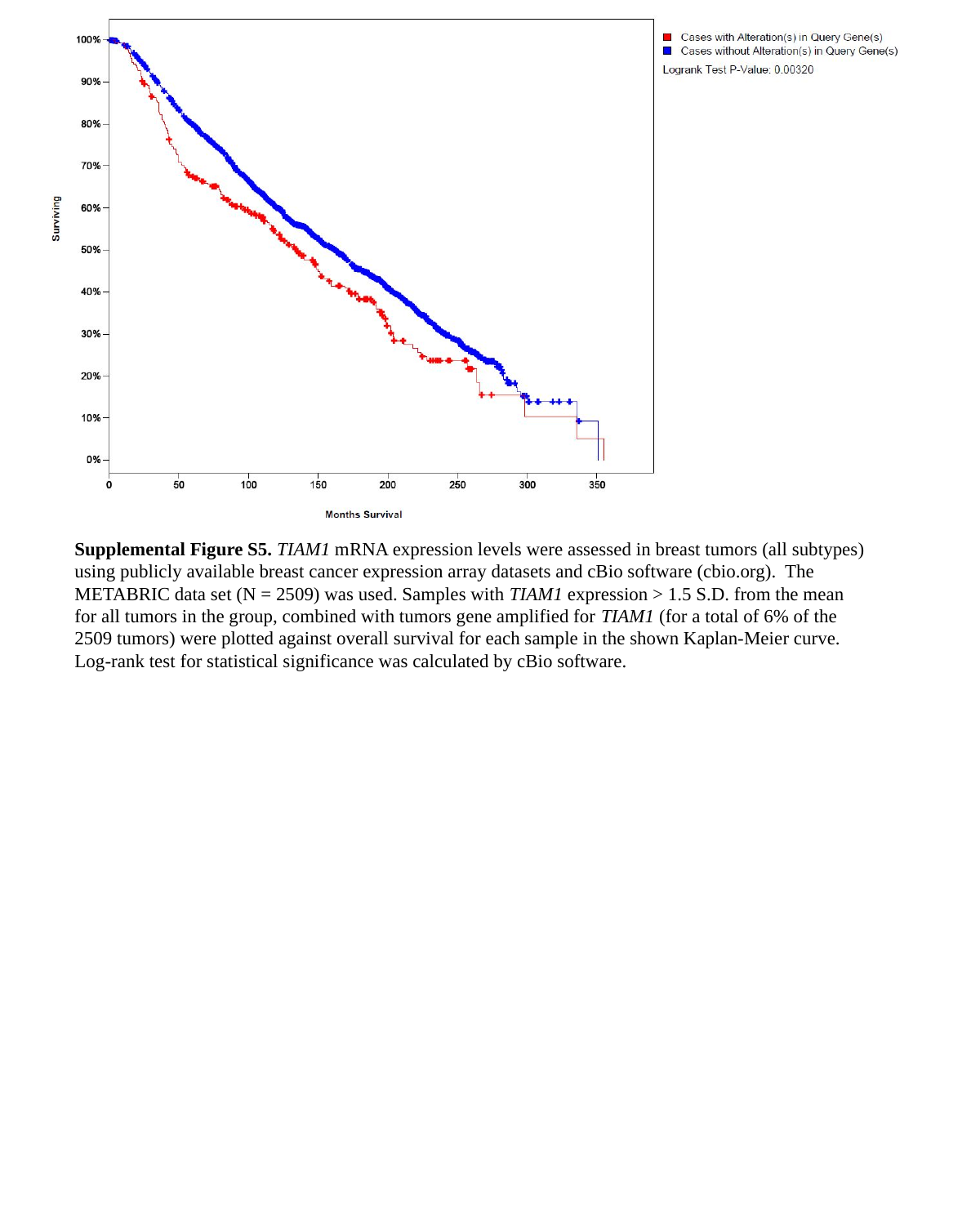

Supplemental Figure S5. TIAM1 mRNA expression levels were assessed in breast tumors (all subtypes) using publicly available breast cancer expression array datasets and cBio software (cbio.org). The METABRIC data set (N = 2509) was used. Samples with TIAM1 expression > 1.5 S.D. from the mean for all tumors in the group, combined with tumors gene amplified for TIAM1 (for a total of 6% of the 2509 tumors) were plotted against overall survival for each sample in the shown Kaplan-Meier curve. Log-rank test for statistical significance was calculated by cBio software.

## Slide 6
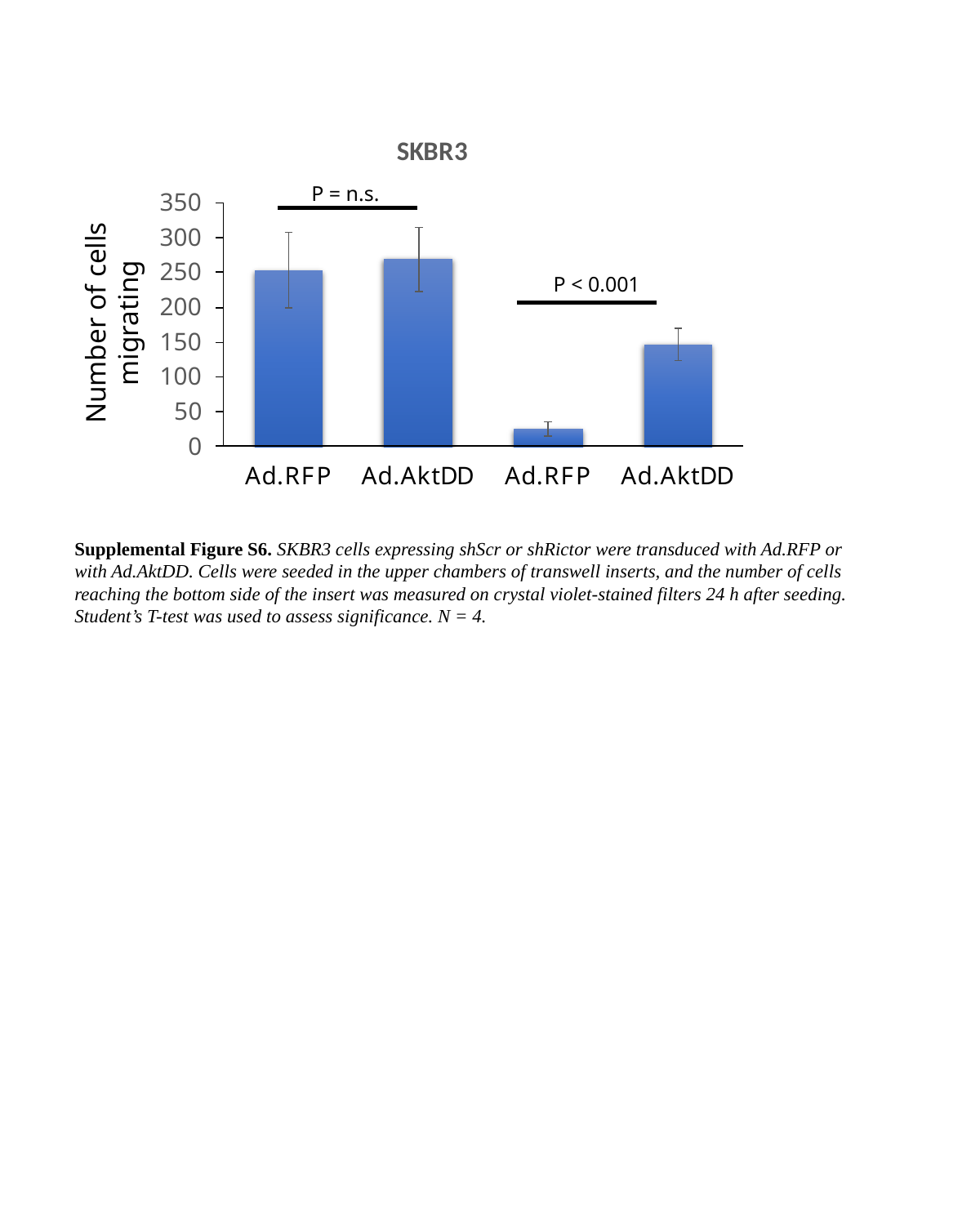

P = n.s.
P < 0.001
Number of cells migrating
Supplemental Figure S6. SKBR3 cells expressing shScr or shRictor were transduced with Ad.RFP or with Ad.AktDD. Cells were seeded in the upper chambers of transwell inserts, and the number of cells reaching the bottom side of the insert was measured on crystal violet-stained filters 24 h after seeding. Student’s T-test was used to assess significance. N = 4.
